# Engineering Exosomes for Cancer Therapy

**DOI:** 10.3390/ijms18061122

**Published:** 2017-05-24

**Authors:** Katie E. Gilligan, Róisín M. Dwyer

**Affiliations:** Discipline of Surgery, Lambe Institute for Translational Research, National University of Ireland Galway (NUIG), Galway H91 YR71, Ireland; K.GILLIGAN3@nuigalway.ie

**Keywords:** exosomes, cancer, therapy, microRNA, electroporation, lipofection, viral

## Abstract

There remains an urgent need for novel therapeutic strategies to treat metastatic cancer, which results in over 8 million deaths annually worldwide. Following secretion, exosomes are naturally taken up by cells, and capable of the stable transfer of drugs, therapeutic microRNAs and proteins. As knowledge of the biogenesis, release and uptake of exosomes continues to evolve, and thus also has interest in these extracellular vesicles as potential tumor-targeted vehicles for cancer therapy. The ability to engineer exosome content and migratory itinerary holds tremendous promise. Studies to date have employed viral and non-viral methods to engineer the parent cells to secrete modified exosomes, or alternatively, to directly manipulate exosome content following secretion. The majority of studies have demonstrated promising results, with decreased tumor cell invasion, migration and proliferation, along with enhanced immune response, cell death, and sensitivity to chemotherapy observed. The studies outlined in this review highlight the exciting potential for exosomes as therapeutic vehicles for cancer treatment. Successful implementation in the clinical setting will be dependent upon establishment of rigorous standards for exosome manipulation, isolation, and characterisation.

## 1. Introduction

Metastasis is the leading cause of cancer related deaths worldwide, thus novel tumor-targeted therapeutic strategies are urgently required. In 2012, there was an estimated 14.1 million new cancer cases globally, with approximately 8.2 million deaths [1]. While cell-based therapies are promising, significant safety issues remain in relation to activation of a toxic host immune response [2]. There has been much progress in regards to gene therapy in recent years with many different vectors investigated [3]. Exosomes contain a range of proteins, lipids, mRNAs and microRNAs (miRNA) [4] and are naturally taken up by cells in order to deliver these contents to recipient cells [5,6,7,8]. Increased understanding of this process has spurred a rapid expansion of research in this field, investigating the use of exosomes as vehicles to transport therapeutic microRNAs, proteins or drugs directly to tumor cells. Exosomes provide a relatively stable environment for the therapeutic agent of choice, have the potential to be modified to improve cell specific homing, and have the ability to fuse with the plasma membrane of cells allowing therapy to directly enter the cell. Allogenic exosomes are thought to allow for decreased immune response, potentially overcoming one of the main challenges of cell-based therapies [9]. The ability to engineer exosome content and migratory itinerary holds tremendous promise. Studies to date have employed viral and non-viral methods to engineer the parent cells to secrete modified exosomes, or alternatively, to directly manipulate exosome content following secretion. While the majority of studies have focused on engineering exosome content, there is also growing interest in mechanisms to modify the exosome surface to facilitate targeted uptake by tumor cells, while sparing healthy cells. Understanding factors controlling packaging of exosome content, release, and uptake by target cells will be key to successful translation of this exciting approach to the clinical setting.

## 2. Direct Modification of Isolated Exosomes for Cancer Therapy

The majority of studies investigating drug loading into exosomes have used non-viral methods such as liposomes, incubation, and electroporation (Table 1). Electroporation takes advantage of the exosomes porous structure. The drug of choice is placed in suspension with the exosomes, which then have an electric field applied. This allows the drug to enter the exosomes through pores created by the electric field. The concentration of exosomes ranges from 0.07–0.5 µg/µL per electroporation, with successful uptake seen in many studies at varying voltages [4]. However, limitations have been encountered when loading exosomes using this method, including precipitation of siRNA, and poor efficiency of DNA transfer [10,11]. Another non-viral method by which exosomes can be modified is by simple incubation, employed in many studies investigating exosomes altered to carry drugs. Either the parent cells are incubated with the drug or the exosomes are first isolated and then incubated with the drug; however, the molecular size of the drug must be small enough to penetrate the exosome membrane [4]. Several studies investigating exosomes in cancer research use chemically-based methods, e.g., Lipofection, to modify the exosomes. Lipofection is commonly used because it is highly reproducible, effective and simple for transient expression of the transfected material [12]. However, disadvantages include a low transfection efficiency, and, in the case of cell manipulation, there is a high dependence on cell division [13]. A paper published recently examined the optimal way of loading exosomes before administration into a tumor-bearing model [9]. The potential of macrophage-derived exosomes to deliver paclitaxel (PTX) to multiple drug resistant cancers was assessed (Table 1). Exosomes were loaded by incubation, electroporation and sonication using exosomes released by macrophages. In vitro, it was found that sonication resulted in sustained drug release and high loading efficiency when compared to the other methods. More importantly, however, it was discovered that exosomes increased the cytotoxicity of PTX more than 50 times. In vivo, complete co-localization of airway-delivered exosomes with lung metastases was demonstrated with a significant inhibition of growth of metastases seen in the treatment group [9].

Dendritic cells (DC) have the unique capability of inducing both primary and secondary immune responses. Therefore, exosomes derived from DCs have been extensively investigated for their contribution to immune modulation, resulting in three early phase clinical trials investigating their potential as cell free vaccines for cancer [14,15,16] (Table 1). DC exosomes were first investigated as a cell free vaccine in 1998 [17], when it was discovered that DCs secrete exosomes that express T-cell costimulatory molecules and functional Major Histocompatibility Complex (MHC) classes I and II. The study compared the injection of DCs into a tumor-bearing model to cell-free injection of exosomes derived from immature DCs. In the group receiving exosomes alone, a potent effect was observed, with delayed tumor growth as well as complete regression of the tumor in 60% of mice. DC exosomes pulsed with tumor peptides (GM-CSF and IL-4) were also employed in vivo, which resulted in suppression or eradication of the established tumor. The results of the study spurred several other investigations into the potential of using exosomes as a cell free vaccine [17,18,19]. This then progressed to a Phase I clinical trial in 2005, investigating the potential of DC derived exosomes as a vaccination against metastatic melanoma [15]. The trial highlighted the safety of exosome administration as well as the feasibility of clinical scale exosome production. Patients received four cycles of therapy, with minor responses seen in four out of the 15 patients. As it was a Phase I trial, tolerance and toxicity were the primary focus, with no adverse effects observed in any patients. The trial concluded that the hypothesis deserved further investigation in the patient setting. Subsequently, the authors investigated a potential alternative effector mechanism and received positive results [20]; however, no follow up clinical trial investigating the amended vaccine has since been reported.

A further Phase I clinical trial investigating DC exosome-based immunotherapy for advanced non-small cell lung cancer was published in 2005 [16]. This study showed similar results, in that the therapy was well received with some stability of the disease seen; however, progressive clinical trials have not been published since. In 2007, a Phase I clinical trial focused on using exosomes derived from autologous ascites with/out granulocyte-macrophage colony-stimulating factor (GM-CSF) for colorectal cancer therapy. This study was larger than the other clinical trials with almost 40 patients completing the study. The group that received exosomes in combination with GM-CSF demonstrated a tumor specific anti-tumor cytotoxic T lymphocyte (CTL) response. Although the study had a promising outcome, there was no follow up trial further investigating the therapy [14]. Recently, a group investigated the potential of DC exosomes in vitro as a treatment for breast cancer and found that the incorporation of DC exosomes by tumour cells increased T-cell activation when compared to the control group [21].

Many drugs cannot cross the blood brain barrier (BBB), restricting efficacy of many therapies for metastatic disease. Therefore, it was investigated as to whether exosomes derived from brain endothelial cells could carry a drug across the BBB [22]. Exosomes were isolated and incubated with doxorubicin and paclitaxel. The fluorescent label rhodamine 123 was used to monitor exosome migration following injection into the cardinal vein of zebrafish. This revealed that the exosomes efficiently delivered drugs across the BBB into the brain. When applied to a brain cancer model, it was found that there were fewer labelled cancer cells in the brain of the treated group compared to the control group. This shows that engineered exosomes can efficiently deliver cancer drugs across the BBB and may potentially be used for treatment of brain cancers or metastases [22].

Although investigators continue to look at the potential for direct modification of native exosomes, the majority of research in this field now focuses on engineering the parent cell to secrete genetically modified exosomes.

## 3. Genetic Engineering of Exosomes

Due to the small size of exosomes, many investigators engineer the donor cell followed by the isolation of exosomes containing the gene or drug of interest [4]. Exosomes have been modified to carry a range of contents (Table 2). Gene delivery is achieved using either non-viral or viral methods, and different methods have been investigated attempting to optimise the most efficient loading of the exosomes. Exosomes leave the cell through the endosomal pathway, and viruses have been known to hijack this pathway and use it for their own benefit [23]. By manipulating this process, exosomes can be engineered to carry a gene or drug of interest using a virus to “load” the exosomes. However, the donor cell needs to be chosen carefully as it is known that exosome characteristics and contents will reflect the cell of origin. Many studies have used tumour derived exosomes; however, little is known about the role exosomes play in the premetastatic niche, and whether these exosomes will have potential negative effects [24,25]. There are two main types of viral vectors used: retroviral and adenoviral. Retroviral vectors (including Lentiviral) transduce cells with a high efficiency and can achieve sustained transgene expression; however, they carry a risk of insertional mutagenesis. Adenoviral vectors can be used to transduce both dividing and non-dividing cells to allow transient expression; however, humans have developed an immune response to the adenoviral gene, which limits the re-administration of the same vectors [26]. A core benefit to using exosomes isolated from these cells is that it is then not necessary to directly administer virus to patients.

### 3.1. Modifying the Surface of Exosomes

The surface of exosomes has been modified to support tumour-targeted delivery of contents, which will be critical in the setting of metastatic disease. Exosomes derived from a human embryonic kidney cell line (HEK293) were engineered to express the GE11 peptide and microRNA Let-7a (Table 2). GE11 binds to Epidermal Growth Factor Receptor (EGFR), which is displayed by a number of tumours of epithelial origin. The exosomes were labelled with the near-infrared dye Xenolight DiR (1,1′-dioctadecyltetramethyl indotricarbocyanine Iodide) and injected intravenously into tumour bearing mice. The migratory itinerary of exosomes was then monitored using an In Vivo Imaging System (IVIS) both in vivo and ex vivo, and it was found that three times more GE11 exosomes reached the tumour when compared to the control group. This work then progressed to determining whether the GE11 exosomes could deliver a tumour suppressive miRNA, Let-7A, directly to the tumour. Exosomes were administered to mice via tail vein injection. Tumour growth was measured following four sequential injections of 1 µg exosomes weekly for four weeks, with effective suppression of tumour growth observed in the Let-7a treated group when compared to the control group [27].

Surface modification has also been employed with exosomes derived from murine immature dendritic cells. The cells were engineered to express the exosomal membrane protein Lamp2b fused to αγ integrin-specific iRGD peptide [28]. Isolated exosomes were then loaded with Doxorubicin (Dox) using electroporation, with an encapsulation efficiency of up to 20%. The engineered exosomes were then injected intravenously and shown to deliver Dox to tumours, resulting in inhibition of tumour growth [28].

Bellavia et al. [29] also engineered HEK293T cells to express Lamp2B, in this case in conjunction with the IL3-receptor, which is overexpressed in Chronic Myeloid Leukemia (CML). IL3-Lamp2B (IL3L) exosomes were loaded with either Imatinib or BCR-ABL siRNA. CML bearing mice were then treated with either IL3L Imatinib exosomes or IL3L BCR-ABL siRNA exosomes twice weekly for three weeks. Animals that received the IL3L exosomes had improved tumor targeting, when compared to exosomes without the surface modification. Although not significant, a marked reduction in tumor size was also observed in the Imatinib group, with slower tumor growth seen in the BCR-ABL siRNA animals [29]. Together, these studies support the approach of modifying exosomes with a targeting ligand on the surface to allow direct targeting of tumors [27,28,29].

Tumour Necrosis Factor (TNF)-related apoptosis-inducing ligand (TRAIL) is one of a family of “death receptors”, capable of inducing apoptosis in cancer cells but not normal cells. A group recently transduced k562 leukaemia cells with human TRAIL to create TRAIL+ secreted exosomes. In vitro TRAIL+ exosomes were found to induce apoptosis in melanoma and lymphoma cell lines [30]. In vivo, the exosomes homed to the site of the tumour with accumulation in the lungs, liver and spleen. In tumour bearing mice, it was found that the engineered exosomes induced necrosis and vessel damage in the myeloma and melanoma tumour groups, but no significant reduction in tumour volume was observed. However, the lymphoma group had a significant reduction of tumour growth when compared to the control groups [30]. It was suggested by the authors that the reasoning for the results observed were that only a fraction of the administered exosomes actually homed to the site of the tumour, with large amounts becoming trapped in major organs. The study showed success in sensitive cancers; however, the method of administration needs to be optimized to allow the exosomes to reach tumour.

Yuan et al. [31] also generated TRAIL+ exosomes, in this case derived from engineered Mesenchymal Stem Cells (MSCs). TRAIL+ exosomes were demonstrated to effectively induce apoptosis in a range of cancer cell lines including lung, pleural mesothelioma, renal, breast and neuroblastoma. The effects seen could be blocked through caspase activity inhibition or TRAIL neutralisation. While no toxicity was observed in control healthy cells, TRAIL+ exosomes were capable of inducing apoptosis even in TRAIL—resistant cancer cells, an effect that was further enhanced using a CDK9 inhibitor. This promising data warrants further investigation in vivo.

### 3.2. Genetic Engineering of Exosome Content

Exosomes naturally carry genetic material and much research is now focused on taking advantage of this, with many cancer-based studies investigating exosomal therapy using microRNAs (miRNAs) (Table 2). MiRNAs are small (19–22 nts) non-coding RNAs, that target mRNAs usually through binding to the 3′ untranslated region. Once bound, the miRNAs either silence or degrade the RNA of interest thus preventing translation to protein [43]. MiRNAs contribute to most biological functions and were discovered to play a role in cancer in the early 2000s [44]. MiRNAs have since been demonstrated to play both tumour suppressing and oncogenic roles [45,46]. Exosomes act like a shield keeping the miRNAs intact and fully functional when transferred to recipient cells [43]. Therefore, there has been a rapid expansion in the number of studies investigating the potential of exosome-mediated delivery of miRNAs or anti-miRNAs as a therapy. Exosomal miRNAs have been investigated in relation to breast cancer in vitro. O’ Brien et al. [32] found that miR-134 was downregulated in breast tumours and played a role in controlling Hsp90; therefore, the potential to use the miRNA as a tumour suppressor in vitro was determined. An invasive breast cancer cell line was modified to overexpress miR-134 and secreted exosomes were then isolated. Exosomes enriched with miR-134 reduced Hsp90, cellular invasion and migration in recipient breast cancer cells, and also enhanced sensitivity to anti-Hsp90 drugs. Bovy et al. [33] employed miR-503 enriched exosomes isolated from endothelial cells and demonstrated impaired tumor cell proliferation and invasion in vitro. Both studies highlighted promising potential of exosome encapsulated tumour suppressing miRNAs for the treatment of cancer.

Munoz et al. [40] focused on knockdown of an oncomiR, miR-9, which has been found to effect sensitivity to chemotherapy. The miRNA has been found to be increased in glioblastoma multiforme (GBM) cells that are resistant to temozolomide (TMZ). Therefore, it was suggested that by decreasing the expression of miR-9, the cells would become more sensitive to TMZ. MSCs were transduced with anti-miR-9 and then cultured indirectly with GBM cells separated by a transwell membrane (0.4 µm). MSCs transferred the anti-miR-9 to GBM cells through exosomes, with no transfer observed when the release of exosomes was blocked using 2.5 μmol/L manumycin A. Further analysis showed that anti-miR-9 effectively sensitized the GBM cells to TMZ with an increase in caspase activity and cell death [40].

Several in vivo studies have also been performed with promising results (Table 2). THP-1 macrophages were transfected with miR-143 and secreted exosomes subsequently isolated. The exosomes were intravenously injected into mice bearing colon cancer daily for two days at 1–5 × 10^5^ exosomes per injection. It was found that miR-143 expression was significantly increased in the tumour, kidneys and serum of animals. Effective suppression of tumour growth was also observed [34].

MiR-146b has been found to silence EGFR and to reduce invasion and motility of glioma cells; however, expression of this miRNA is lost in the majority of glioma tumours. Katakowski et al. [36] investigated miR-146b as a potential tumour suppressor in glioma. Mesenchymal Stem Cells (MSCs) were transfected with miR-146b using electroporation, and secreted exosomes administered via intra-tumoural injection into a rat model of glioma (50 µg total protein), five days after tumour implantation. Rats that received the miR-146b exosomes displayed a significant reduction in tumour volume after only one injection when compared to the control groups.

The impact of exosome encapsulated miRNAs on tumor chemosensitivity has also been investigated. Hepatocellular carcinoma (HCC) has been found to display a resistance to chemotherapies such as 5-fluorouracil (5-FU) and doxorubicin. Loss of miR-122 in patients with HCC has been linked with disease metastasis and poor prognosis [35]. Over expression of miRNA-122 in a mouse model was found to inhibit tumourigenic properties and also sensitize the cells to chemotherapies such as sorafenib and doxorubicin. It was then investigated whether Adipose derived MSCs (AMSCs) could be modified to express miR-122 and secrete exosomes enriched with the miR to restore HCC chemosensitivity. Exosomes containing miR-122 were administered intra-tumourally to BALB/c nude mice with HepG2 tumors, combined with sorafenib treatment. One intra-tumoural injection of exosomes significantly reduced the tumour weight and volume when compared to the control group, showing that miR-122 AMSC exosomes could increase HCC cell sensitivity to chemotherapy [35].

### 3.3. Genetically Modified Exosomes for Immune Modulation

As previously described, it has been extensively investigated as to whether native exosomes could be used as a potential cancer vaccine; however, these studies did not genetically modify the exosomes. Dai et al. [41] incorporated IL-18 into exosomes using a recombinant adenovirus. These exosomes were found to be capable of promoting PBMC proliferation as well as the secretion of Th1 cytokines, chemoattracting T and DC Cells in vitro when investigated in relation to colon cancer. Another group investigated the effect of IL-2 and its antitumor effects [42]. EL-4 cells that were previously engineered to over-express Ovalbumin were transfected again to overexpress IL-2. The engineered exosomes were then isolated from these cells and injected into tumour bearing mice. The engineered exosomes could induce an antigen-specific Th1 polarized immune response and CTL more efficiently, which resulted in significant tumour growth inhibition [42].

Cho et al. [37] used mouse cell lines (CT26 and TA3HA) where exosomes were modified to express human MUC1 (hMUC1) to see if they could stimulate an immunologic response in vivo. The exosomes were isolated and confirmed to be expressing elevated hMUC1 before being intradermally injected into BALB/c, H-2d mice. In this case, the mice were injected with tumour cells three weeks following exosome administration, and tumour size was monitored. The engineered exosomes were shown to stimulate immune cell activation as well as suppressing growth of hMUC1 expressing tumours.

Another study employed exosomes from A549 lung cancer cells transfected with the Ras-related protein RAB27a [38]. BALB/c nude mice received subcutaneous injections of RAB exosomes two weeks before being injected with A549 cells. The results showed that pre-immunization with exosomes significantly inhibited tumour growth in vivo. The study also examined the effect of the exosomes on pre-established tumours, with inhibitory effects seen on tumor growth [38]. In a separate study investigating mouse melanoma, mice were treated with α-galactosylceramide/ovalbumin loaded exosomes [39]. The exosomes were found to increase T-cell tumour infiltration, decrease tumour growth and increase median survival when compared to the control groups. These studies combined highlight an important role for engineered exosomes in immune modulation in the cancer setting, which can potentially be harnessed for disease therapy.

## 4. Discussion

The potential to use exosomes as therapeutic agents is an exciting and rapidly evolving field of research, with tremendous potential to impact the prognosis of patients with metastatic cancer. However, there remain considerable challenges to be overcome, and the speed of progress must be tempered with rigorous experimental standards and reporting to have a meaningful impact. The studies outlined in this review highlight the variety of approaches and therapeutic agents that can be employed for modification of exosomes, but further understanding of the fundamental biology of these extracellular vesicles is required to support optimal utility. A basic current challenge exists in relation to the nomenclature and definition of exosomes. Regardless of the isolation method employed, all exosome samples are heterogeneous in nature, as other extracellular vesicles have similar characteristics and overlap the defining size of exosomes (30–120 nm). Investigators are now encouraged to use the more broad terminology of “Extracelllular Vesicles (EVs)” to reflect the heterogeneity of samples, while providing specific details of the experimental protocols employed to isolate and then characterise the EVs. This will support meaningful interpretation of data reported and reproduction of experiments to validate findings.

EVs hold tremendous promise in the therapeutic setting considering their relative ease of isolation and manipulation of both the contents and migratory itinerary. The small size allows EVs to cross barriers that cells cannot, and initial data suggests that EVs maintain an immune privilege that may be advantageous. Understanding the differences in biology of EVs derived from different sources, and their migratory itinerary, will be key to progress.

Until recently, the majority of studies reported EV yield or experimental dosing in microgram (Protein) amounts. It is now known that this is not a suitable surrogate for EV quantity, as individual EVs can contain varying amounts of protein. In addition, the majority of the investigations performed to date have a short study timeline (up to 28 days), which must be extended as we move forward.

As our knowledge continues to evolve rapidly, it will be critical to have transparent reporting and sharing of information. To this end, an international consortium has established “EV-TRACK” for just this purpose, to support standardisation of EV research through systematic reporting on the biology of EVs and the methods used for isolation [47]. The consortium created a scoring paradigm, EV-METRIC, for publications in the EV field. This metric highlights nine components of information that are required to support thorough interpretation and reproduction of experiments in the field. This includes the specific parameters associated with the isolation method (e.g., ultracentrifugation g-force, rotor), analysis of a minimum of three EV-enriched proteins, and both close up and wide-field electron microscopy images. Successful implementation of EVs for the treatment of cancer in the clinical setting will be dependent upon establishment of rigorous standards for EV manipulation, isolation, and characterisation.

## Figures and Tables

**Table 1:** Studies employing exosomes without genetic modification for the treatment of cancer.

Exosome Source	Setting	Therapy	Tumour	Study Outcome	Reference
Macrophages (RAW 264.7)	In Vivo	PTX/DOX	Lung Mets	Exosomal PTX preferentially accumulated in cancer cells	[9]
Ascites-derived	Clinical trial	AEX alone or AEX + GM-CSF	Colorectal	AEX + GM-CSF was safe, nontoxic, tolerable, and induced a beneficial tumour-specific anti-tumour CTL response	[14]
Dendritic cells	Clinical trial	MHC Class II peptides	Melanoma	Large scale exosome production was feasible and exosome administration was safe and well tolerated	[15]
Dendritic cells	Clinical trial	MAGE (tumour antigens)	Lung	Therapy well tolerated with some experiencing long term stable disease and activation of immune effectors	[16]
Dendritic cells	In Vivo	IL-4 + GM-CSF	Breast	Eradication/suppression of growth of pre-established tumours in a T-cell dependant manner	[17]
Dendritic cells	In Vivo	MHC Class I	Melanoma	MHC Class I restricted CD8+ T-cell expansion and differentiation	[18]
Dendritic cells	In Vivo	CpG Adjuvant	Melanoma	Combination of exosomes and TLR 3 + 9 triggered efficient MHC-restricted CD8+ T-cell responses	[19]
Dendritic cells	In Vivo	DC-Exo alone	Melanoma	DC-Exo promoted IL-15Rα- and NKG2D-dependent NK cell proliferation and activation which resulted in anti-metastatic effects	[20]
Dendritic cells	In Vitro	DC-Exo alone	Breast	Incorporation of DC-Exo by tumour cells increased ability to activate T-cells for a more effective response	[21]
Brain endothelial cells	In Vivo	rhodamine 123, PTX, DOX	Brain	Exosome delivery allowed DOX and PTX to cross the BBB which resulted in cytotoxicity against U-87 MG cells	[22]

Abbreviations: AEX—Ascites-derived exosomes; GM-CSF—granulocyte-macrophage colony-stimulating factor; CTL—cytotoxic T lymphocyte; PTX—Paclitaxel; Dox—Doxorubicin; IL—Interleukin; MAGE—Melanoma-associated antigen; DC—Dendritic Cell.

**Table 2:** In vivo and in vitro studies using modified exosomes for cancer therapy.

Exosome Source	Setting	Therapeutic Agent	Tumour Model	Study Outcome	Reference
Kidney cells (HEK293)	In Vivo	GE11 peptide + Let-7a	Breast	Tumor targeted delivery of Let-7a suppressed tumour growth	[27]
Dendritic cells	In Vivo	Lamp2b fused to αγ iRGD peptide + DOX	Breast	Significant inhibition of tumour growth, with no overt toxicity	[28]
Kidney cells (HEK293T)	In Vivo	Lamp2b IL3 + Imatinib or BCR-ABL siRNA	Chronic Myeloid Leukemia	IL3L surface improved tumor targeting. IL3L-Imatinib: reduced tumor size; IL3L BCR-ABL siRNA: slower tumor growth	[29]
Breast cancer (Hs578T)	In Vitro	miR-134	Breast	Increased miR-134 significantly reduced STAT5B, Hsp90 and Bcl-2 levels resulting in reduced cellular proliferation	[32]
HUVECs	In Vitro	Pre/anti-miR-503	Breast	Increased miR-503 decreased both proliferation and invasion.	[33]
Leukemia cells (THP-1)	In Vivo	miR-143	Colon	Increased miR-143 levels in tumours resulted in suppression of growth.	[34]
AMSCs	In Vivo	miR-122	Hepatocellular carcinoma	Cancer cells were rendered sensitive to chemotherapy through miR-122 expression	[35]
MSCs	In Vivo	miR-146b	Glioma	Intra-tumoural exosome injection significantly reduced tumour volume	[36]
Mouse colon (CT26) & breast (TA3HA)	In Vivo	hMUC1	Colon	Tumour size was reduced by MUC-1. CT26-MUC-1 higher dose and TA3HA-MUC-1 lower dose showed best results.	[37]
Lung cancer (A549)	In Vivo	Rab27a	Adenocarcinoma	Immunization with Rab27a significantly inhibited tumour growth, with similar results seen in pre-established tumours	[38]
Mouse Bone Marrow Cells	In Vivo	α-Galactosylceramide	Melanoma	Induced an early iNKT-cell response, dendritic cell, MZB cell activation as well as NK- and T-cell activation and proliferation	[39]
MSCs	In Vitro	Anti-miR-9	Glioblastoma mutliforme (GBM)	Reverse expression of miR-9 sensitized the GBM cells to TMZ which increased cell death and caspase activity	[40]
Colon (LS-174T)	In Vitro	IL-18	Colon	Exo/IL-18 can chemoattract DCs and T cells which induces IFN-γ augmented release of IL-2 and promoted T-cell proliferation	[41]
Mouse thymoma (E.G7-OVA)	In Vivo	Ovalbumin, IL-2	Thymoma	Induced antigen specific Th1-polarized immune response and CTL more efficiently resulting in tumour regression	[42]
Leukemia (K562)	In Vivo	TRAIL	B Lymphoma; Melanoma	Inhibition of tumour growth was seen in both groups, although not significantly in the melanoma group	[30]
MSC	In Vitro	TRAIL	Variety of cancer cell lines	Induction of apoptosis in range of cancer cell lines, including some TRAIL resistant cells. Effect enhanced through use of CDK9 inhibitor.	[31]

Abbreviations: Dox—Doxorubicin; AMSC—Adipose derived Mesenchymal Stem Cells; HUVEC—Human Umbilical Vein Endothelial Cells; TRAIL—Tumour Necrosis Factor related apoptosis-inducing ligand.
